# Contemporary perspectives on Lacanian theories of psychosis

**DOI:** 10.3389/fpsyg.2013.00350

**Published:** 2013-06-28

**Authors:** Jonathan D. Redmond

**Affiliations:** School of Counseling, Australian College of Applied PsychologyMelbourne, VIC, Australia

**Keywords:** psychosis, Lacan, Miller, Verhaeghe, actual neurosis, ordinary psychosis, stabilization

## Abstract

In contemporary Lacanian psychoanalysis, Verhaeghe's theory of actualpathology psychopathology in psychosis and the Millerian idea of “ordinary psychosis” provide diverging conceptual approaches to psychosis. In this paper, the two approaches to psychosis are examined with a particular emphasis on “mild psychosis” and compensatory mechanisms. Despite the shared focus on similar clinical phenomena, particularly body disturbances, these two theories provide different explanations of psychosis. Verhaeghe's theory of psychosis is a synthesis of Lacanian theory, Freud's idea of actual neurosis and psychoanalytic attachment concepts. Moreover, these ideas are situated in the “schizophrenia/paranoia dichotomy” an important heuristic device utilized in clinical practice with psychosis. In contrast, the Millerian field of ordinary psychosis aims to broaden the idea of psychosis by reviving the idea of “mild psychosis” and the different forms of stabilization possible in psychosis. Clinicians adapting the idea of ordinary psychosis aim to rethink pivotal Lacanian concepts—“untriggered” psychosis and stabilization—beyond the scope of the schizophrenia/paranoia dichotomy. Although the idea of ordinary psychosis requires further development, it promise greater utility than Verhaeghe's model, as it provides a broader and more nuanced approach to the complex vicissitudes of triggering and restitution in psychosis.

## Introduction

In Lacanian psychoanalysis psychosis continues to be an important focal point for new theoretical developments driven by clinical experience. Two important new developments have emerged over the past decade that provide contrasting approaches to Lacan's oeuvre and the theorization of psychosis. Paul Verhaeghe, in *On being normal and other disorders:a manual for clinical psychodiagnostics* provides a fascinating approach to psychosis through his synthesis of Lacanian psychoanalysis with Freud's theory of actual neurosis and psychoanalytic attachment theory research. His theory of psychosis is important as it addresses forms of psychosis “without symptoms.” That is, he engages with aspects of psychosis not easily contained by contemporary psychiatric nosology such as, psychosis without delusions and hallucinations, untriggered psychosis and body disturbances such as hypochondriasis. Moreover, he provides a specific treatment rationale for cases of psychotic disturbances that fall roughly into the schizophrenic spectrum. In contrast, Jacques-Alain Miller's engagement with the “later Lacan” informs his theoretical approach to the emerging field referred to as “ordinary psychosis.” The term ordinary psychosis provides an epistemic category—as opposed to a new nosological entity—for clinicians to address a series of theoretical problems linked to decompensation and stabilization often encountered in the treatment of psychosis (Miller, 2009; Grigg, 2011). Both projects are a response to contemporary clinical phenomena, often referred to as “new symptoms,” and the perceived limitations to aspects of Freudian and Lacanian theory. Despite this, these two approaches differ substantially from each other, and as such, provide significantly different views of psychosis and the possibilities of treatment. In a recent interview, Verhaeghe made several criticisms of the field of ordinary psychosis. First, he thinks that Miller's idea has “little if anything to do with psychosis in the classical Lacanian sense” (Verhaeghe, 2011, p. 20). And second, he believes that the term is likely to create confusion for non-psychoanalytically trained clinicians and is therefore likely to be unhelpful. As such, important theoretical and practical debates are at stake between these two approaches.

The aim of this paper is to develop a clearer sense of the conceptual divide between Verhaeghe's theory of psychosis and the Millerian field of ordinary psychosis. I provide an overview of these two diverging approaches and indicate the conceptual advantages of the Millerian idea of ordinary psychosis. I claim that Verhaeghe's theory has several limitations. First, there *appears* to be conceptual ambiguity in his theorization of foreclosure and drive regulation in psychosis. Second, the theory of foreclosure, which underlies Lacan's idea of psychotic structure, is relatively under-theorized. And finally, by focusing almost exclusively on the schizophrenia/paranoia dichotomy, he utilizes a reductive idea of psychosis. In contrast, I claim that the field of ordinary psychosis provides a more nuanced engagement with psychosis. My reading of ordinary psychosis emphasizes how foreclosure is intimately connected to three practical elements of treatment: the onset of psychosis, subsequent triggering events and stabilization^1^. I show how the field of ordinary psychosis broadens the contemporary psychiatric acceptation of psychosis by affirming that psychotic phenomenology are rich, complex, subtle and that symptom severity has no necessary connection to the idea of psychotic structure. Although Verhaeghe would undoubtedly be in agreement with this last point, I contend that the Millerian category of ordinary psychosis provides a more nuanced account of psychotic phenomena and psychosis. To achieve this, I begin by summarizing Lacan's theory of psychotic structure to anchor the discussion of the differences between Verhaeghe and Miller.

## Lacan's theory of psychotic structure

In Lacanian theory, the neurosis/psychosis distinction remains central to how analysts conceptualize clinical practice^2^. While Lacanian nosology has strong links to modern psychiatry, the Freudian theory of the unconscious is used to articulate the different mechanisms underlying neurosis and psychosis^3^. For Lacan, both psychosis and neurosis are situated vis-à-vis the subject's relation to the Other and in particular, the signifier known as the “Name-of-the-Father.” The Name-of-the-Father is associated with an array of functions linking the subject with the Other; these include castration, symbolic identifications, desire, and the installation of the proper name. Fundamentally, the Name-of-the-Father is as signifier regulating the unconscious, in part, through creating a structural limit (i.e., castration) to the subject's capacity for drive related jouissance. In neurosis, the subject's relation with the Other is mediated by the signifier, the Name-of-the-Father, via symbolic identification. Hence, different forms of neurosis such as hysteria and obsessional neurosis are constituted by repression, a mechanism driven via the subject's identification with the signifier the Name-of-the-Father. Although foreclosure, like repression, is a form of negation (i.e., the subject negates this signifier), they are not the same; they have significantly different consequences in the formation of psychic structure.

Lacan asserts that a psychotic structure emerges from the foreclosure of the Name-of-the-Father. Thus, the foreclosure of the Name-of-the-Father is the central mechanism in psychosis and differentiates psychosis from neurosis. His classical theory of psychosis conceptualizes psychic structure by focusing on the subject's position in the symbolic order via the Name-of-the-Father. The mechanism of foreclosure, developed by Lacan from Freud's texts on psychosis (1911, 1918), is a form of negation that is contrasted with repression. In neurosis, repression occurs when signifiers are turned away from consciousness into the unconscious. In psychosis, foreclosure is a unique form of negation such that the subject *never affirms* the existence of the signifier, the Name-of-the-Father. Consequently, this signifier is foreclosed and is never registered in the subject's symbolic universe. Instead of signifiers being repressed, a mechanism that presupposes the judgment of existence (Lacan, 1953), the foreclosure of the signifier the Name-of-the-Father leaves a hole in the Other (Lacan, 1958).

In psychosis, the foreclosure of the signifier entails that the subject may encounter a hole in the symbolic at pivotal junctures in subjective experience. The rupture in the signifying chain occurs when the subject is unable to signify aspects of their existence along the axes of metonymy and metaphor. Problems with metonymy underlie many of the language disturbances encountered in psychosis such as “loose associations” and a break down in syntax. Here, the one-to-one linkage between signifiers is interrupted. In contrast, the absence of the paternal metaphor in psychosis causes the subject's link to language to differ significantly when compared to the neurotic subject. The absence of an anchoring signifier, the Name-of-the-Father, may produce radical disturbances to subjectivity, as there is literally no way of representing specific aspects of subjective experience. As metaphor functions to designate the position of the subject in the signifying chain, which is intimately linked to the question of meaning and identity (Vanheule, 2011), then the absence of the signifier, the Name-of-the-Father, can have significant consequences; this is particularly evident in the subject's relation to sexuality and the drives. I return to in these points later in this paper.

Lacan's theory of psychosis utilizes the idea of “unitary psychosis” by supposing that the mechanism of symbolic foreclosure is a necessary and sufficient condition for a psychotic structure. Unitary psychosis is characterized by the claim that there is only one fundamental mechanism underlying all non-organic psychosis (Berrios and Beer, 1994), despite significant variations in symptomatology, and with the recognition that distinct sub-groups of psychosis—schizophrenia, paranoia, and melancholy—do exist. This is important to note because for Lacan, the emphasis on unitary psychosis is augmented by what classical psychiatry calls the “schizophrenia/paranoia” dichotomy.

In classical psychiatry, the schizophrenia/paranoia distinction emerged from the clinical observation of symptomatology in psychosis. On the one hand, the schizophrenia/paranoia dichotomy is a nosological distinction. For example, in schizophrenia psychotic phenomena are often complex and variable; unsystematised delusions, confabulations, hallucinations, social withdrawal, and a range of disorganized behavior such as vegetative states, body disturbances and incoherent cognitive processes, may be encountered (Laplanche and Pontalis, 1973; Sadock and Sadock, 2003; Verhaeghe, 2004). In contrast, in paranoid psychosis, a delusion may be the only overt symptom; and in certain cases, even this may be subtle and difficult to detect. On the other hand, the schizophrenia/paranoia dichotomy supposes that psychotic phenomena have a tendency to evolve from an abstract and disorganized state into a systematized form (Meisser, 1981; Hriso, 2002; Stanghellini, 2009). Thus, clinicians have long observed the progressive, evolutionary tendency of psychosis; the disorganization inherent to classical schizophrenia symptomatology has the tendency to evolve into a systematized delusion of the paranoid type. In these instances, the “deficit” symptoms and disorganization of schizophrenia will disappear with the emergence of systematized delusional phenomena. Freud's analysis of these phenomena was groundbreaking as it provided a treatment orientation for analysts working with psychotic patients and remains a central pillar to the subsequent theorization of psychosis in the Lacanian field.

In Freud's (1911) major work on psychosis, the schizophrenia/paranoia dichotomy was conceptualized in terms of decompensation and recovery. His engagement with the Schreber case was significant because he asserted that delusional phenomena had stabilizing effects when compared to the disorganization of classical schizophrenia. For Freud, the formation of a delusion was an attempt at recovery because it correlated with the subject's reengagement with the world. In schizophrenia, radical disorganization is painful and the individual is often unable to engage in the most basic social relations and activity in the world. In contrast, in paranoia the formation of a delusion is often linked with mitigating effects—not only do the classical schizophrenia symptoms disappear but the formation of a delusion also correlates with the subject's reengagement with others, albeit in a modified form (Freud, 1911). In Lacan's classical theory of psychosis articulated in the 1950s, both the schizophrenia/paranoia dichotomy and Freud's thesis of restitution are pivotal to his approach to the question of psychotic structure. In following Freud's reasoning, Lacan states that the distinction between schizophrenia and paranoia is essential to his own theorization of psychosis^4^. However, in Lacan's classical theory of psychosis, paranoia as opposed to schizophrenia was the focal point of his work in the 1950s^5^. Through introducing the idea of a “delusional metaphor,” Lacan transformed the ideas of progressive systematization and restitution^6^. However, an overemphasis on the paranoid spectrum of psychosis and on Lacan's account of the mechanisms encountered in paranoia, has meant that non-delusional forms of stabilization, particularly in the schizophrenic end of the spectrum, are poorly understood.

## Case vignette

Emphasizing the schizophrenia/paranoia dichotomy entails that stabilization is viewed primarily in terms of paranoia and the formation of a delusion. In contrast, clinicians using the term “ordinary psychosis” have observed that body phenomena may be linked to the stabilization of an individual in the absence of a delusion. The second part of this paper explores these themes. I discuss a case vignette illustrating the centrality of the body symptoms in the stabilization of psychosis. I use this case as a starting point for outlining Verhaeghe's theory of psychosis that attempts to integrate empirical research with Freudian and Lacanian theory. I then develop a critique of his position and introduce important issues linked to the field of ordinary psychosis relating to the ideas of untriggered psychosis, stabilization, and body symptoms.

In “The use of metonymy in a case of psychosis,” Deffieux (2000) offers a theory of metonymy as the framework for conceptualizing the symptomatisation in psychosis.

The case of “Murielle” provides insight into how invasive and painful body phenomena become transformed into a localized symptom that appears to have a stabilizing function. An important feature of the case concerns the connection between body phenomena, symptoms and her unique history: specifically, Murielle's medical history concerning scoliosis can be traced to both invasive psychotic phenomena and the emergence of a body symptom.

Murielle initially presented to a clinic with severe body pains: wrist and ankle pain and body inflammations traversed her body. Medical evaluations do not show any organic basis for the phenomena. Murielle's body pains have a complex aetiology. At age eleven, she was diagnosed with scoliosis and as part of the treatment she wore a corset to bed every night until the age of eighteen. Her psychotic symptoms began as a teenager shortly after encountering her ill father in hospital; she was shocked and overwhelmed to find her father very weak, in pain and barely conscious when receiving prostate treatment. Murielle was hospitalized for severe body pains a few days after visiting her father. Several years later, she again experienced psychological difficulties after finishing the medical treatment with the corset: persecutory voices, in the form of “hearing” thoughts and whispers of other students, emerged shortly after the cessation of treatment.

Although Deffieux considers the diagnosis of hysterical neurosis and conversion disorder, both he and the treatment team arrive at the diagnosis of *paranoia with hypochondriasis* due to the invasive nature of jouissance upon the body and the existence of paranoid traits. Deffieux states that the onset of body disturbances is clearly linked to her father's illness and suggests that the influx of invasive body jouissance at this time correlates with the breakdown of her image of the father, and can therefore be theorized in the context of an “undialectised” imaginary identification between father and daughter. Moreover, he hypothesizes that vicissitudes of psychotic phenomena are linked to the corset's removal; he states that “*jouissance*, no longer circumscribed by the corset, found a new localization in the Other, more precisely in the gaze of the Other” (Deffieux, 2000, p. 153). Here, a clear transition from body phenomena to a nascent delusional structure that is expected in paranoia occurs. However, at the beginning of the treatment, the *invasive jouissance moves from the gaze of the Other back to the body*. In summary, he states that “it is interesting to note the transition of this mobility of jouissance which moves from the body fitted up with its train of suffering, to the delusional interpretation of the Other's gaze and then returning in the body in the oblique way of hypochondria” (2000, p. 153).

Although Deffieux assumed a diagnosis of psychosis with a paranoid structure, Murielle's mode of stabilization was not through the production of a delusional metaphor. As the invasive jouissance returned to her four limbs not supported by the corset, Deffieux suggests the aim of treatment is to reduce the intensity of “pain from her body and of finding substitutes for the previous corset” (2000, p. 154). Instead, invasive body jouissance was mobilized into the elaboration of a body symptom via a signifying series. Here Deffieux states:

The theoretical guide in this case was fairly simple: we noted, then followed and accompanied the subject's metonymic thread, giving it all its therapeutic value, which was to delimit the invasive jouissance. What was the metonymical series in this case? One must begin with the ritual of the water basin from when she was a child, recognize the value of the corset when she was an adolescent, and from that follow the ritual of washing her feet and hands and which moved on, always following through this series, to delimiting herself little by little to wetting her feet and then to wrapping her hands in a damp flannel and her toes in cotton bandages. The last in the chain came to her following a conversation, through the advice to use a “hydrating cream.” This last minimal link was nonetheless enough for her. The pain completely disappeared, but she retained a peculiar, precautionary way of walking, as if she were stepping on egg shells (2000, p. 156).

This case provides a useful reference point for discussing both Verhaeghe's ideas on psychosis and the field of ordinary psychosis; while it is in no way “paradigmatic” of ordinary psychosis it raises issues concerning the onset of psychosis, body disturbances and non-delusional forms of stabilization. I return to these themes later in the paper. I now discuss these issues in more detail by examining Verhaeghe's theory of psychosis.

## Verhaeghe's theory of psychosis: actualpathology and psychopathology

Verhaeghe's engagement with Lacan's classical theory of psychosis emphasizes the importance of the schizophrenia/paranoia dichotomy. However, unlike Lacan, Verhaeghe develops this dichotomy by drawing on Freud's theories of symptom formation in actual neurosis and psychoneurosis and, he also draws on empirical research in attachment theory. On the one hand, Freud's distinction between the actual neuroses and psychoneuroses underlies the differing symptomatology in neurosis and psychosis; on the other, by drawing on theories of mirroring in attachment theory, he aims to provide a developmental mechanism for understanding the aetiology of symptomatology in schizophrenia and paranoia. In summary, Verhaeghe's approach to the schizophrenia/paranoia dichotomy consists of three distinct theoretical approaches: Lacan's idea of psychotic structure, Freud's theory of symptom formation in actual neurosis and psychoneurosis, and finally, psychoanalytic attachment theory research conducted on mirroring behavior between parent and infant.

Verhaeghe coined the phrase the “actualpathology/psychopathology continuum” for examining symptom formation across the schizophrenia/paranoia dichotomy^7^. His theory of actualpathology is based on Freud's theory of anxiety equivalents in the actual neuroses^8^. Actualpathological states are essentially anxiety equivalents that correlate with the clinical phenomena described by Freud in cases of actual neurosis. The actual neuroses refer to a cluster of clinical phenomena, in both neurosis and psychosis, where anxiety directly affects the body: disturbances include migraines, panic attacks, free-floating anxiety, gastro-intestinal irritation, and other somatic phenomena^9^. One useful aspect of Verhaeghe's theory is that he utilizes actualpathology in *neurosis and psychosis* when speaking about contemporary clinical issues and DSM nosology. For example in neurosis, actualpathology may be useful for considering panic disorder, PTSD, somatization, and borderline personality disorder (BPD). However, this is not necessarily a simple undertaking as diagnoses such as BPD, which often include *transitory psychotic states*, could be also be viewed in terms of a psychotic structure and untriggered psychosis (Maleval, 2000) a point I return to later. Nevertheless, Verhaeghe's views on actualpathology provide a useful framework for considering certain aspect of *DSM-IV-TR* diagnosis and contemporary clinical issues.

Freud originally conjectured that clinical phenomena belonging to actual neuroses were linked to the direct manifestations of anxiety. He used the term “anxiety equivalents” to describe them; anxiety equivalents emerge when endogenous body excitations cannot be transformed from a physical state into a psychological phenomena^10^. Anxiety equivalents emerge in the body and tend to be directed to a particular region creating variable physiological disturbances to the normal function of the organ^11^. The key difference though, between anxiety equivalents that emerge in the body and psychoneurotic symptoms, such as hysterical conversion symptoms, is that the latter are formations of the unconscious while anxiety equivalents are an “abnormal employment of libido” that are not formed through the mechanisms of unconscious displacement and condensation (Freud, 1916, 1917). Thus, anxiety equivalents are literally meaningless as there are no repressed ideas and chains of associations underlying the symptom.

Following on from Freud, Verhaeghe claims that the emergence of anxiety equivalents can be traced to the libido and the drives. However, he modifies Freud's (1893) theory that actual neurosis is the result of undischarged libidinal tensions and instead proposes that the subject's endogenous drive excitation has not been sufficiently regulated by the Other (Verhaeghe, 2004). For Verhaeghe, actualpathology indicates that in the subject's developmental history, the drives were not sufficiently modulated by the attachment figure. He states,

The causal factor of actualpathology … lies in the fact that the subject's internal drive excitation is not—or is insufficiently—answered by the other. The transition from (a) to A through which the Other supplies an answer and sets the secondary processing into motion does not occur, with the result that the initial arousal turns into anxiety and even into separation anxiety (Verhaeghe, 2004, pp. 300–301).

Verhaeghe states that actualpathology is characterized by a failure of the other to adequately modulate endogenous drive tension, particularly during infancy. Drive tension refers to the innate endogenous body tensions requiring regulation; however, due to innate infantile helplessness, the other is the locus through which drive tensions are regulated. Verhaeghe draws on Lacan's “object *a*” when describing endogenous drive tensions that the subject is unable to regulate through symbolization: the object *a*, closely linked to Freudian drive theory, the erogenous zones and Klein's part object, produces an impasse in symbolization akin to trauma^12^. He uses the term “structural trauma” when referring to the subject's incapacity to symbolize, and therefore modulate, the drive tensions; anxiety generated via the subject's encounter with the object *a* constitutes a structural trauma, a universal feature of psychic reality, and *anxiety equivalents* develop when the Other does not adequately regulate this endogenous drive tension^13^.

Thus, as the human infant is born into the world in a state of radical helplessness and is thus dependent on the Other, the function of regulating endogenous drive tensions depend on the subject's relation with the Other. In actualpathology, drive tensions are not sufficiently transformed into psychical states and therefore remain at the level of the real; if this occurs, anxiety equivalents predominate in the clinical picture. Moreover, his theory also relies heavily on a developmental paradigm; he states that attachment figures—the Other—will determine whether the subject is sufficiently able to regulate the drive^14^.

In Verhaeghe's (2004) theory of actualpathology, attachment figures are primarily responsible for the regulation and modulation of endogenous drive tension. He claims that the subject's inability to modulate the drive is understood in terms of a disturbance to the attachment system and specifically, the mirroring dynamics between the subject and the other. He situates this failure of the mirroring function using Lacan's theory of the mirror stage (other) and the Other^15^. Verhaeghe states:

To put it in Lacanian terms, something went wrong during the mirror stage that is, the period where identity formation starts in combination with drive regulation. It seems as if the contemporary Other—meaning the parents, but also the symbolic order—is failing more and more in taking on his/her mirroring function. The result is that the child does not develop a psychological, meaning a representational way, of handling his drives and the accompanying arousal. Moreover, identity formation as such is hampered as well (2007, p. 9).

He argues that actualpathology is characterized by anxiety equivalents, rather than formations of the unconscious, as the attachment figure has failed to produce sufficient signifiers for the subject to modulate body arousal (Verhaeghe, 2004). However, the disturbance to the mirroring relation between the subject and the Other, which constitutes the mechanism underlying actualpathology, is elaborated using the dynamics of infant/caregiver interactions derived from psychoanalytic attachment research (Fonagy et al., 2002, 2003; Bateman and Fonagy, 2004, 2006).

Verhaeghe contends that a developmental paradigm is required to conceptualize the transformation of drive tensions into a representation psychical form. He asserts that ideas about metallization, particularly those of Fonagy et al. (2002), provide a theory outlining the mechanism and aetiology of actual neurosis. He argues that “in light of Freud's conclusion that, in actual neuroses, the process of psychic representation is lacking, attachment theory permits us to assume that actual neuroses originate from a deficient mirroring” (Verhaeghe et al., 2007, p. 1335). He claims that contemporary psychoanalytic attachment theory provides a model for locating the developmental aetiology for the actual neuroses using the mirroring dynamics evident in early infant/caregiver interactions. Thus, the primary mechanism informing his theory of actualpathology, which aims to explain how endogenous drive arousal becomes excessive to the point of traumatism, is based on a failure in the mirroring relation between the subject and the Other.

Verhaeghe's theory of actualpathology then is based on the idea of deviant mirroring styles identified by researchers in psychoanalytic attachment theory. He claims that the disturbances to the mirroring relation outlined by Fonagy et al. (2002) result from the Other's failure to modulate the subject's endogenous drives tension. Here, the primary defense against the drive fails, due to the way in which “the Other will mirror the tension of the subject and/or the way the subject interprets this mirroring” (Verhaeghe, 2004, p. 190). This claim is derived from Fonagy et al.'s theory of “deviant” mirroring styles; they contend that “if the caregiver mirrors the baby's emotions inaccurately or neglects to perform this function at all, the baby's feelings will be unlabeled, confusing, and experienced as unsymbolised and therefore hard to regulate” (2002, p. 426). As he believes that deviant mirroring styles constitutes the mechanism underlying certain symptom constellations in psychosis then it will be useful to provide a brief outline of how these ideas inform his work.

According to Fonagy et al. (2002) “deviant mirroring” styles encountered in the attachment relationship results in metallization deficiencies and affect disregulation. They claim that deficits in metallization correlate with affect regulation disturbances: if the capacity for mentalisation is compromised, affective regulation disturbances and other disorders are likely to develop. According to Fonagy et al. (2002) mentalisation occurs via the operation of mature ego functions developed from the nurturing environment of a secure attachment relationship. Mentalisation refers to preconscious and conscious representation capacities utilised in negotiating inter-subjective relations and for the regulation of affect. Mentalisation is linked with the development of a sense of self; the capacity to attribute intentional states, beliefs, goals, desires and emotions to the self and the other, is also a developmental achievement, and it is the emergence of these capacities that correlates with the capacity for affect regulation. Affect disregulation occurs when an individual is unable to modulate emotions through self-soothing: instead, the subject experiences affects as labile, unpredictable and disorganizing. Although mentalisation deficiencies and affect disregulation emerge when neglect, abuse, or chronic misattunement are present in the infant/caregiver dyad, the authors claim that these events can be theorized in terms of deviant mirroring dynamics. Bateman and Fonagy (2004) claim that there are at least two types of deviant mirroring styles and both have potentially traumatizing effects:

Mirroring would be expected to fail if it is either too close to the infant's experience or too remote from it. If the mirroring is too accurate, the perception itself can become a source of fear, and loses its symbolic potential. If it is absent, not readily forthcoming, or contaminated with the mother's own preoccupation, the process of self-development is profoundly compromised (2004, p. 35).

In the first example, where the parent's mirroring is analogous with the infant's affect, the mirroring is too realistic due to a high degree of similarity. Here, the negative affect is categorically congruent; the parent mirrors the infant's affect state too realistically and does not “mark” the representation of the affect state. There are several consequences to this. First, the infant may identify with the caregiver's emotional disregulation; consequently, the infant's consistent exposure to the caregiver's negative affect is both alienating and disorganizing. Second, the infant will not create secondary representations of its primary affect state, as no “anchoring” emerges between affect and representation through the parent's mirroring. Anchoring refers to the associational link between secondary representations and the primary affect; here, a deficiency of self-perception will emerge in conjunction with an affect regulation disturbance. Third, negative affect will be externalized onto the other. Finally, realistic mirroring in an unmarked form escalates the infant's negative affect leading to traumatism: the lack of maternal containment and processing of negative affect escalates the negative affect to the point of dissociation and splitting of the ego (Fonagy et al., 2002).

In contrast, in the second form of deviant mirroring, where the mirroring is absent, there is a lack of category congruence between the affect and its secondary representation. In this instance, the mirroring performed by the caregiver is too dissimilar from the infant's primary affect state. There is marked, but inaccurate, mirroring of infant's primary affective state. Mirroring is partially effective; the infant develops secondary representations anchored to the primary affect state. However, these representations are incongruent with the affect; therefore, a distorted sense of the affect states may ensue^16^.

Verhaeghe draws directly on these theories of “deviant” mirroring styles in his approach to certain clinical phenomena in psychosis. He claims that psychosis, particularly cases featuring body phenomena without hallucinations and delusions, is characterized as the Other's failure to modulate the subject's drive. In Verhaeghe's description of this failure, it is clear that Fonagy et al.'s (2002) first description of deviant mirroring styles—where mirroring is too congruent, marking is absent, and negative affects have increased—underlies his approach. This is significant for his approach to the treatment of psychosis because Verhaeghe believes the failure of Other to adequately modulate the subject's drive will be repeated in the transference; in actualpathology the transference will likely be characterized by rejection, guilt, appeal and refusal in the subject's relation with the Other (2004, 2007). For Verhaeghe, the failure of the Other to adequately mirror and hence modulate the subject's drive tension produces a structural trauma in the formation of the psyche. Here Verhaeghe states:

Actualpathology has been characterized as that group of disorders where the subject remains stuck in primary development: the Other doesn't answer, or failed to answer sufficiently. As a result, the initial (un)pleasure and anxiety, together with their somatic anxiety equivalents, persist in an unelaborated form. The resulting disorders center on somatization and anxiety, accompanied by reactive avoidance behavior. No processing occurs in the representational order, hence the absence of a fundamental fantasy and symptoms (2004, pp. 351–352).

When discussing actualpathology in psychosis, Verhaeghe claims that endogenous drive tensions make a demand on the subject and the only way to respond is through anxious preoccupation. He states that body disturbances are most frequently encountered in the *schizophrenia* spectrum of psychosis indicating that “the first logical moment is the moment of onset, namely, the actualpathological confrontation with (a) in the psychotic structure” (2004, p. 445). However, in his synthesis of actualpathology and psychosis, anxiety equivalents are the focal point of his discussion of body phenomena. In actualpathology in psychosis, the clinical presentation is often characterized by hypochondriacal phenomena and panic disorder; secondary symptoms such as hallucinations and delusions may be altogether absent. For Verhaeghe, hypochondriacal complaints and intrusive body phenomena indicate that drive arousal has not been psychically represented. He claims that hypochondriasis emerges from the impossibility of *psychically representing* somatic drive arousal; in a psychotic structure, the failure of the Other to adequately modulate the subject's drive tension governs the emergence of body phenomena such as hypochondriacal symptoms. In actualpathology, there is no substitution by signifiers and no symbolization; thus, the development of a symptom articulated via a chain of signifiers is not evident and the disturbance remains in the form of an anxiety equivalent.

He then advances the idea that the stabilization of disorganizing body phenomenon in schizophrenia is best achieved via the construction of a delusion; this engenders a level of psychical organization that uses secondary defenses and a network of signifiers to bind drive tensions in the form of a delusional construction. Verhaeghe (2004) states that the exploration of the original “failed” relation between the subject and the Other in the transference must occur for the subject to construct a delusion. He states:

In actualpathologies, the primary aim of the treatment is the restoration or even the installation of the primary relation between the subject and the Other through the therapeutic relation. It is this that will enable the subject to build up a secondary elaboration and, through the transferential relation, embed the original body arousal into signifiers, enabling symptoms to be constructed. To put it correctly, one must begin with an exploration of the original relation between the subject and the Other (with emphasis on separation anxiety) and on the remaining signifiers that is, the minimal original inscriptions of the somatic in the Symbolic-Imaginary order. Rather than subject analysis, the therapeutic goal here is subject amplification (2004, p. 309).

Verhaeghe's conceptualization of psychosis and stabilization recapitulates the schizophrenia/paranoia dichotomy. He advocates that one treats anxiety equivalents encountered in actualpathology by the elaboration of a delusional construction in therapy. He calls this process “subject amplification.” Verhaeghe states that in actualpathology, interpretations have no effect because there is nothing to interpret –as there is no meaning to anxiety equivalents, the phenomena do not have a “metaphorical” structure and therefore a repressed or hidden meaning cannot be elucidated^17^. Disturbances encountered in actualpathology are anxiety equivalents *not* substitutive symptom formations; for Verhaeghe (2004), to interpret actualpathological phenomena as meaningful, which supposes a metaphorical structure, is technically incorrect and is likely to induce guilt in the subject. Conversely, subject amplification orientates clinical intervention; the treatment aim, and the focus of therapeutic intervention, is to develop the “minimal signifiers marking the body” into the form of a signifying construction. Through a naming process unique to the subject's own articulation of signifiers, the repetition of the original failed subject/Other relation with a guaranteeing Other (the therapist) in the transference transforms anxiety inducing body arousal into symptom formations. Here, the treatment aim of developing secondary representational processes requires specific techniques irreducible to interpretations. The therapist needs to focus on providing a supportive and name-giving relationship that is oriented to an empathic engagement with the “here-and-now” (Verhaeghe, 2004). He claims that the first step is the installation of a primary relation between the subject and the Other in the context of a secure relation with the Other; the therapeutic relationship becomes the foundation for building the secondary representational processes required to manage drive arousal that was hitherto experienced as overwhelming. The provision of a secure relation with the Other in transference relation is the first logical step in the movement toward secondary processing of the drive. However, as the subject's relation to the Other in the actualpathological position is characterized by a failure to modulate drive tensions, the therapist's intervention will likely be experienced as failure, as never being good enough; in essence, a testimony to the historical rejection from the Other that will be necessarily repeated in the transference.

According to Verhaeghe (2004), therapeutic engagement with psychosis in the actualpathological position necessitates alterations to classical analytic technique. The recapitulation of developmental history has important consequences for the role that constructions have in the direction of the treatment; constructions emerge through the mirroring and naming function that the therapist assumes due to the subject's experience of developmental failure with the Other. In this sense, subject amplification is the process where signifiers are installed to represent the original somatic arousal, establishing secondary representational processes that facilitate the emergence of “classical symptoms” (Verhaeghe, 2004). This recapitulates Fonagy's idea of attuned mirroring linking affect with signifiers evident in the secure attachment system between parent and infant. In psychosis, the construction of a delusion entails a distinct shift in the processing of anxiety: anxiety equivalents, localized in the body, are transformed into signal anxiety taking the form of the delusional metaphor. Although delusional systems can be persecutory, over time the subject's active role in producing a delusional construction will have a stabilizing function^18^. For Verhaeghe, the delusion is not only a form of recovery, but also, an indication for the direction of treatment in cases of actualpathology and psychosis. Moreover, once the delusional system has evolved to a point of relative stability, the invasive jouissance becomes contained and encapsulated in delusions that are discrete formations that do not dominate the subject's life entirely. The emergence of symptom formations from the actualpathological position entails the progressive development and stabilization of a delusional system. This trajectory reflects his ideas regarding how psychosis tends to naturally progress, which emphasizes the schizophrenia/paranoia dichotomy, and thus to constructing a delusion in the treatment of psychosis. However, there are a series of problems with his account.

## Actualpathology and the field of ordinary psychosis

In Verhaeghe's theory of psychosis there appear to be difficulties distinguishing between the effects of foreclosure from the Other's failure to modulate the subject's drive tension. For example, in hypochondriasis where body phenomena are predominant, the impossibility of representing drive arousal with the signifier could be an effect of foreclosure, rather than the difficulty in modulating the drive. This is because in Lacan's theory of psychosis (1958; 1993), the mechanism of foreclosure is linked to disturbances to phallic signification; although such disturbances have diverging manifestations, one important feature is the subject's inability to represent that is, to signify, fundamental elements of experience such as sexuality and embodiment (Sauvagnat, 2000; Vanheule, 2011). Thus, although he correctly claims that as the psychotic subject does not have access to phallic signification, the psychotic has a significantly different experience of the body and jouissance when compared to the neurotic: however, his theory of actualpathology does not clearly differentiate them. Thus, in arguing that the “subject's perplexity is an expression of the impossibility of answering the drive's jouissance” (Verhaeghe, 2004, p. 446), this impossibility could be due, in his approach, to either the effects of symbolic foreclosure *or*, to the failure of the Other to modulate the subject's drives. Consequently, it is difficult to separate the mechanism of deviant mirroring from the foreclosure of the Name-of-the-Father in his explanation of the failure of the Other to modulate the subject's drive. Hence, the inclusion of deviant mirroring styles to explain mild psychosis remains to be clarified due to the under-developed theorization of foreclosure and deviant mirroring styles.

Following from this emphasis on the failure of the other to modulate the subject's drive, his description of endogenous drive arousal in psychosis focuses extensively on anxiety. As a consequence, the effects of foreclosure on the subject are minimized. Thus, the central problem in psychosis as articulated in Lacanian theory—the difficulty in regulating jouissance due to foreclosure of the Name-of-the-Father—is in Verhaeghe's account, shifted primarily to *anxiety*. Although anxiety in psychosis is of importance (Sauvagnat, 2005), the primacy of symbolic foreclosure, and its clinical effects, should be paramount; that is to say, anxiety in psychosis should be oriented to foreclosure and difficulties in regulating jouissance. For example, in hypochondriasis, although anxiety is clearly evident, the more pertinent issue concerns the subject's inability to regulate invasive jouissance due to the absence of the signifier, the Name-of-the-Father. Hence, the feeling of perplexity that so often accompanies hypochondriacal phenomena (Sauvagnat, 2000; Porcheret et al., 2008; Stanghellini, 2009) may be attributable to the subject's inability to create meaning at a specific juncture. This is a significant issue because in Lacan's theory of psychosis a complex array of clinical phenomena may emerge as a consequence of the foreclosure of the Name-of-the-Father (Miller, 2009), and, in following Freud's thesis concerning loss and restitution in psychosis, psychotic phenomena need to be oriented to the question of onset and stabilization.

Verhaeghe does not adequately discuss in detail the three distinct theories of stabilization in Lacan's theory of psychosis. Stabilization in psychosis is pivotal to Lacan's work and he approaches this issue from at least three separate angles: imaginary identification, the formation of a delusion, and the sinthome (Grasser, 1998). Although Verhaeghe briefly mentions the “as-if” phenomena and the theory of sinthome, both are linked to the vague notion of a “prodromal” psychotic phase and are not adequately developed. Moreover, his claim that the theory of sinthome is virtually equivalent to the construction of a successful symptom in neurosis is also opaque: his discussion is very brief and the allusion to the sinthome, as being equivalent to the construction of a symptom, is not sufficiently expanded or explained. Thus, other forms of stabilization, such as for cases with no obvious onset, are alluded to as a “successful psychosis” but are given sparse attention.

Finally, another problem in Verhaeghe's theory of actualpathology is that he adopts the position that the construction of a delusion is the only method to stabilize a subject after the onset of psychosis. Thus, he privileges the construction of a delusion as the modus operandi of stabilization and doubts that other forms of stabilization in psychosis are possible post onset. He states that the aim of treatment in psychosis is the construction of a systematized delusion:

The psychotic subject doesn't have the luxury of a conventional language and hence of a conventional, shared solution for the real. This is why the psychotic must create a private solution, namely, a delusion. That this delusion is the psychotic's solution—perhaps even the only possible one—has not been recognized in today's approaches (Verhaeghe, 2004, p. 431).

On the one hand, he is right to state that the delusion, as a form of recovery, has not been sufficiently recognized in contemporary psychiatric theories of psychosis. On the other, his emphasis of the schizophrenia/paranoia dichotomy creates a reductive view of psychosis as the disorganized phase of acute schizophrenia becomes the basis for theorizing stabilization according to the formation of a delusional metaphor. His theory of stabilization in psychosis aims to transform the body phenomena, often encountered in schizophrenia, into paranoia. Thus, although his conceptualization of this process is unique, it is, nevertheless, a recapitulation of the schizophrenia/paranoia dichotomy and Freud's thesis that the delusion is a form of recovery. Hence, his theory does not develop anything *new* in terms of understanding the different mechanisms of stabilization in psychosis. I now focus on the field of ordinary psychosis and show how this approach to psychosis addresses some of the limitations evident in Verhaeghe's theory of psychosis.

## The field of ordinary psychosis

The term “ordinary psychosis” emerged from a series of discussions on contemporary clinical practice in the *World Association of Psychoanalysis*. The term, coined by Miller, was first used during a series of conferences at Angers, Arcachon, and Antibes (Laurent, 2006)^19^. The term was invented to provide an opportunity for clinicians to discuss “rare cases” where the clinical symptomatology could not easily be situated in the neurosis/psychosis distinction. These rare cases did not follow the “typical” presentation of either a neurosis or a psychosis as the signs and symptoms were vague and therefore difficult to catalogue. Moreover, the mechanisms of repression and foreclosure underlying Lacan's theory of psychic structure in neurosis and psychosis were also difficult to deduce from the clinical picture (Svolos, 2008). Taking psychosis as an example, cases were presented where the clinical picture did not match with the classical symptoms of schizophrenia (cognitive and corporeal disorganization, hallucinations, and language disturbances) or of paranoia (delusions). In addition, although some cases were suggestive of psychosis, the absence of a clear onset of symptoms—a phenomena described by Lacan (1993) and an idea that gained traction in the way clinicians think about psychosis—created additional uncertainty. As the meetings continued it became clear that these rare cases were *more common* than first thought; clinicians realized that difficult to classify cases were being seen on a regular basis. An important result of these meetings was the reengagement with Lacan's work on psychosis, though in a format that went beyond both his classical theory of psychosis and theorization of paranoia; instead, Lacan's ideas on “untriggered psychosis” and the “suppletion” became central to further exploration of the category of psychosis, as used in the Lacanian field. Miller's theorization of ordinary psychosis emerges from this context.

Miller's (2009) comments on ordinary psychosis focuses on diagnostic uncertainty. For Miller, the clinical problem of diagnostic uncertainty, which was an important issue to emerge from the conferences, should be addressed in the neurosis/psychosis distinction. His statements on the idea of ordinary psychosis can be viewed as an attempt to respond to diagnostic uncertainty in clinical practice. Specifically, his answer to this problem is to make psychosis a *default* position and in doing so, it is clear that he aims to utilize a broad idea of psychosis—whether this moves beyond Lacan's classical theory of psychosis is a subject to debate and an issue I return to later. The trajectory of his argument is worth noting. First, he states that ordinary psychosis does not denote a new nosological category, but instead, provides an approach to theorizing psychosis. He states,

You say “ordinary psychosis” when you do not recognize evident signs of neurosis, so you are led to say it is a dissimulated psychosis, it is a veiled psychosis. A psychosis that is difficult to recognize as such, but which I infer from various small clues. It's more of an epistemic category than an objective category. It concerns our way of knowing it (2009, p. 149).

Second, if the clinician does not recognize a neurotic structure then they may assume it is a psychotic structure, even if there are *no obvious features of psychosis*. This claim is based on the assumption that *neurosis has a definite structure and that clinicians will be able to recognize it.* He states that neurosis will be characterized by repetition, the clear evidence of castration, and the differentiation between the ego, id, and super-ego (2009), and in the absence of these signs of a neurotic structure, then the analyst may assume he is dealing with a case of psychosis. Thus, one retains the neurosis/psychosis distinction, and by the logic of the “excluded middle,” one is led to conclude psychosis^20^. The logic of the excluded middle refers to cases of diagnostic uncertainty. Miller's response to this problem is that one *should* default to psychosis, given that clinicians will recognize neurosis if it is present. Thus, *doubt* over the diagnosis entails a diagnosis of psychosis.

Two points need to be made here. First, cases of ordinary psychosis, determined from the absence of neurotic symptoms, may be cases of “untriggered psychosis” - this is because a psychotic structure can be assumed to exist despite no obvious psychotic signs and symptoms. This echoes Lacan's claim made in *Seminar III* that “there is nothing that more closely resembles a neurotic symptomatology than a prepsychotic symptomatology” (1993, p. 191). Second, although one might object to Miller on the grounds that it is not as easy to recognize neurosis as he suggests, the notion of ordinary psychosis also includes instances of psychosis that have stabilized post-onset.

In debates concerning ordinary psychosis, the question of what triggers and what stabilizes psychosis is pertinent to cases ranging from mild to severe. Tom Svolos observes,

the question is, are the times between breaks … to be understood as ordinary psychosis? And if we take a category like schizophrenia, do we understand the time between breaks as dormant or quiet or *latent schizophrenia*, or do we understand that as ordinary psychosis? In other words … I think we have a specific, restricted notion of ordinary psychosis … the ordinary psychosis of the banal, where it's very stable and limited and so forth—but then *ordinary psychosis opens up a more general theory of ordinary psychosis against which we can articulate the specific structure of say, schizophrenia or paranoia.* The utility of the concept is the way that it's *broadened our ability to conceptualize psychosis and think about issues of stabilization* in ways that didn't exist in the literature before (2009, p. 165 emphasis added).

These remarks indicate that there are two approaches to ordinary psychosis regarding triggering and stabilization. The first concerns cases where stabilization occurs subsequent to an obvious psychotic break. Although severe psychotic features, such as delusions and hallucinations, may appear subsequent to the triggering of a psychosis for a certain group of psychotics, these symptoms will often attenuate to the point where no obvious evidence of psychosis remains. However, as Svolos notes, the periods of stability between triggering events need to be articulated in terms of stabilization, as opposed to mere phenomenological descriptions (residual phase) or the invention of new diagnostic categories (schizotypal personality disorder)^21^. The second way of speaking of ordinary psychosis, referred to as “banal psychosis” or untriggered psychosis, includes cases where no obvious triggering event has occurred. For Svolos, combining these two perspectives on ordinary psychosis opens a *general notion of psychosis* that focuses on triggering and stabilization. If the idea of ordinary psychosis is oriented toward these two issues—*untriggered psychosis* and a *post-onset stabilized psychosis*—then triggering and stabilization emerge as key features to investigate in this field.

As the investigation of ordinary psychosis is focused, in part, on untriggered psychosis then the idea of “mild” psychosis is an important feature of this field. Milder forms of psychosis are devoid of clinical phenomena such as hallucinations, delusions, mania, and disorganized thought (Laurent, 2006; Svolos, 2008). In their place, a significant number of clinical phenomena remain (Miller, 2009), which are poorly understood by clinicians. Before discussing these issues it must be emphasized that ordinary psychosis has continuity with aspects of classical psychiatry. In one sense, Miller's attempts to “broaden” the category of psychosis vis-à-vis the term ordinary psychosis is, in one sense, merely a call to return to classical psychiatric concepts. He states:

but once you've said that it's ordinary psychosis, it means it's a psychosis, and if it's a psychosis, it may be subjected to classical organizational concepts… Ordinary psychosis must not be a permission to ignore the clinic. It's an invitation to go further than this term (Miller, 2009, pp. 155–156).

For example, Svolos' discussion of ordinary psychosis explicitly engages Bleuler's idea of *latent schizophrenia*, a psychosis without obvious or severe positive and negative symptoms; the conjunction between the two is made as both focus on the idea of mild psychosis^22^. However, the important question for him and other Lacanian theorists using the term ordinary psychosis is whether mild psychosis is considered to be an “untriggered psychosis” or a post-onset stabilized psychosis. I return to these issues later in the paper.

Another parallel between the field of ordinary psychosis and classical psychiatry coalesces around cenesthetic schizophrenia. Cenesthetic schizophrenia shows the primacy of body disturbances in certain forms of schizophrenia, in which typical symptoms of schizophrenia—hallucinations, delusions, and cognitive disorganization—are only transitory phenomena (Huber, 1992)^23^. Huber refers to cenesthetic schizophrenia as a variant of *latent schizophrenia* suggesting “cenesthetic schizophrenia is a schizophrenia that comes to a standstill at its very beginning or develops into pure residual syndromes after one or a few short psychotic episodes” (1992, p. 58). Cenesthetic schizophrenia is a good example of body phenomena in cases of mild psychosis—obvious psychotic phenomena are transitory, disorganized behavior is absent, and diagnosis is difficult due to the brevity of psychotic episodes and the predominance of the residual phase. Interestingly, he echoes Bleuler's claim that latent schizophrenia is probably the most prevalent of all the forms of psychosis.

However, the dominance of DSM focused psychiatry means that these nuanced ideas of psychosis are being ignored and lost in contemporary clinical practice.

The classical psychiatric ideas about psychosis have lost traction for several reasons. The success of the personality disorders, particularly Cluster A and B types, has meant a movement away from the category of mild psychosis. Thus, transitory psychotic states and “unusual” individuals are more likely to be diagnosed with borderline personality disorders and schizoid personality as opposed to psychosis. Changing classical psychiatric ideas on psychosis to create new nosology has created a reductive model of psychosis in contemporary psychiatry. Although the schizophrenia concept in *DSM-IV-TR* (American Psychiatric Association, 2000) is derived from the classical psychiatric tradition of Kraepelin and Bleuler (Sadock and Sadock, 2003) this has been significantly altered and reduced in complexity. This produces a simplistic picture of, and an impoverished clinical engagement with, psychosis. Mullen observes that *DSM-IV-TR* description of psychosis significantly different to the classical psychiatric counterpart:

Bleuler required 95 separate psychopathological phenomena to characterize the schizophrenias, Kraepelin in his final formulation used 75, but *DSM-IV-TR* employs only 30. It is this truncated psychopathology which forms the basis of virtually all characterizations of schizophrenia in today's scholarly literature (Mullen, 2007, p. 114).

Importantly, a reductive model has practical consequence. He states that research surveying psychiatric diagnoses of schizophrenia, in terms of the total number of symptoms counted for each diagnosis of schizophrenia made, shows an average of *4-5 symptoms* used for diagnosis. In this sense, ordinary psychosis provides an opportunity to broaden the category of psychosis, in part, through returning to the classical psychiatric ideas on psychosis. However, from a theoretical perspective ordinary psychosis is centered on Lacan's ideas on untriggered psychosis and the suppletion.

In Lacan's classical theory of psychosis, the idea of an untriggered psychosis highlights the stabilizing function of the imaginary in psychotic structure. An untriggered psychosis, as the name suggests, refers to a psychotic structure without onset. Lacan claimed that in certain cases, imaginary identification may prevent the onset of psychosis: identification with another person provides the psychotic subject with a mechanism of imaginary compensation (Lacan, 1993). He states that narcissistic relations between individuals who *share similar traits* on the imaginary axis constitute a “mechanism of imaginary compensation … for the absent Oedipus complex, which would have given him virility in the form, not of the paternal image, but of the signifier, *the Name-Of-The-Father*” (Lacan, 1993, p. 192). For Lacan, the tendency for imitation evident in narcissistic identification between individuals constitutes a “mechanism of imaginary compensation” that stabilizes the psychotic subject (2004, p. 192). He suggests that in certain cases of *schizophrenia*, imaginary identification functions to stave off psychotic decompensation and used the term, untriggered psychosis, in such cases^24^. An untriggered psychosis may be likened to a broken stool; although minus one leg, a three-legged stool may still function to support a person depending on their weight distribution. However, once the person's weight is shifted above the missing leg, it will collapse, person in tow. Similarly, although imaginary identification functions to keep the person “upright,” psychosis may be triggered, which leads to the collapse of this supportive function.

In the field of ordinary psychosis, it has become clear that imaginary identification can stabilize psychotic structure subsequent to the onset of psychosis and triggering events. Differentiating the onset of psychosis from subsequent triggering events is another important issue in the field of ordinary psychosis. Clearly identifying the onset of psychosis, the *first triggering event*, calls into question the utility of retaining the idea of untriggered psychosis. Although the ideas of an untriggered psychosis and ordinary psychosis both relate to cases of mild psychosis, there is a significant difference between these positions. The idea of untriggered psychosis assumes that the onset of psychosis has not occurred. In contrast, although a case of ordinary psychosis may “look the same” as an untriggered psychosis, the essential difference is that ordinary psychosis may be *post-onset*. For example, an ordinary psychosis case characterized by acute psychosis and subsequent stabilization presented by Ragland (Miller, 2009) demonstrated that despite the history of severe psychotic disturbances, periods of stability were protracted and during these times the subject appeared rather “normal.” The point here is that this case was considered an example of ordinary psychosis (Miller, 2009). In contrast, others claim (Brousse, 2009) that in order to preserve the idea of untriggered psychosis, cases of ordinary psychosis need to be restricted to instances of post-onset stabilisation. In doing so, the distinction between untriggered and post-onset psychosis is maintained. Consequently, despite some “phenomenological” similarity between cases of untriggered psychosis and ordinary psychosis, whether psychosis is pre- or post-onset remains a critical point of distinction.

A problem with the idea of untriggered psychosis concerns the uncertainty in clearly identifying the onset. The investigation of the onset, particularly in milder forms of psychosis, supports the idea that the effects of foreclosure are subtle, difficult to detect, and will vary considerably between cases. As the case of Murielle demonstrated, psychosis can be discreet, without schizophrenic disorganization or a delusion. Although her symptoms emerged at a specific point in time this episode did not constitute a severe onset of psychotic symptoms and she was able to remain in high school after these transient psychotic “episodes.” Thus, one of the problems associated with the idea of an untriggered psychosis is that it produces a propensity to focus on the “first time” that a psychotic episode occurs with the concomitant expectation that a delusional construction will soon follow. While the onset of psychosis is important clinically and attempts should be made to clarify the genesis of psychosis, the onset is often *unverifiable*.

For example, Stevens (2008) raises the issue of whether there has been a clear onset of psychosis in a case featuring a man with fibromyalgia^25^. Stevens claims that the man was psychotic and that the fibromyalgia constituted a *symptom*, as it had several important functions for the subject: the illness provided a proper name, a point of imaginary identification, and a way of localizing invasive body jouissance. Although Stevens states that the patient has an imaginary identification with Christ constituting a “delusion that doesn't make too much noise” (2008, p. 65), he is circumspect on whether there has been an onset of psychosis. He asks, “Is the psychosis in this case triggered or not?” And his answer is that *“it is an academic question*” (2008, p. 65 emphasis added). This response provides a good example of the shift in emphasis evident in the approach to cases of psychosis. If the onset of psychosis is not always discernible then “untriggered” psychosis becomes an unreliable theory. Stevens response corresponds with an emphasis on stabilization and suppletion rather than on the idea of untriggered psychosis and the onset; the key difference is that clinicians are now more interested in identifying small effects of foreclosure indicative of a psychotic structure, as well as the symptomatic responses that may be stabilizing psychotic structure. One may conclude from this that the boundary between an untriggered psychosis and a post-onset stabilized psychosis has become “fuzzy.” Despite this, the idea of untriggered psychosis has not become obsolete. On the contrary, clinical experience testifies to the idea that onset if often acute and emerges without substantial warning or a prolonged “prodromal” phase. Despite this, psychosis is, to use Miller's phrase a “vast continent” and there are many instances where an obvious onset does not occur.

Miller's theory of ordinary psychosis is useful as both the onset of psychosis and triggering events are linked to the more general idea of stabilization and the suppletion. The compensatory make-believe (CMB) Name-of-the-Father is central to his notion of ordinary psychosis: it is ostensibly a supplementary device functioning to cover the hole in the Other, which stabilizes psychic structure. Here Miller states:

For the first time, from a *CMB situation to the opening of a hole*, and it goes on and on, you have a *triggering*. “Multiple breaks” is when you have a repetitive pattern, and it's compensated again and again. *We don't say triggering. We say “triggered” when it happens once* (2009, p. 166 emphasis added).

The CMB Name-of-the-Father is important as it emphasizes the *continuity* between triggering events and stabilization in psychosis. Although the CMB Name-of-the-Father is also applicable to the onset of psychosis and hence cases of untriggered psychosis, *its application is broader* because it encompasses triggering events and stabilization in psychosis, as well. Moreover, as mild cases of psychosis may not exhibit an acute onset then other factors need to be considered. For Miller (2009), a series of “small clues,” referred as the *small clues of foreclosure*, can be useful for conceptualizing mild psychosis. He suggests that subtle instances of foreclosure may indicate “a disturbance to the inmost juncture of the subject's sense of life” (Miller, 2009, p. 154). This phrase, derived from Lacan (1958), can orient the clinician to at least three distinct areas of inquiry concerning the effects of foreclosure: social relations, the body, and subjectivity. Of these, body disturbances are particularly important, as they have been linked to a restitution attempt.

Theorists in the field of ordinary psychosis claim that certain body phenomena in psychosis may have an important stabilizing function. These body phenomena are different to conversion symptoms in hysterical neurosis (Porcheret et al., 2008) and classical hypochondriacal complaints in psychosis (Laurent, 2006)^26^. They are particularly significant because theorists claim that in certain cases the onset of psychosis is followed by *body phenomena* that function to stabilize psychosis. While Lacan's theorization of paranoia is both highly instructive and indispensable to clinicians working with psychosis, theorists have over emphasized the classical theory of psychosis concerning the onset of psychosis and stabilization. In contrast, theorists supporting the idea of ordinary psychosis claim that restitution via symptom formation, as opposed to imaginary identification, is not limited to the formation of a delusion; but rather, a body symptom may effectively short-circuit the trajectory of psychosis described in the schizophrenia/paranoia dichotomy (Gault, 2004; Laurent, 2006; Porcheret et al., 2008)^27^. Theorists claim that for some individuals, a part of the body, experienced as painful, can become a symptom with a *restitutive function*.

One issue central to the field of ordinary psychosis is the claim that body symptoms can have a restitutive function in psychosis. If one accepts the idea that certain body phenomena have a restitutive function then theorizing how the mechanisms of foreclosure and symptomatisation operate in these instances remains to be addressed. Miller's term for suppletion, the CMB Name-of-the-Father, addresses this problem. Two aspects of his argument that support the idea of the CMB Name-of-the-Father are worth noting; the first relates to catatonia and the second concerns the neurosis/psychosis distinction.

Miller's explicit reference to catatonia supports the idea of a supplementary device in psychosis. Catatonia is considered to be the most severe manifestation of psychosis (Cottet, 2000; Declerq, 2004) as it is characterized by radical social withdrawal, body disorganization, and the loss of cognitive functions: in short catatonia indicates the collapse of psychic reality. He (2009) states that if the psychotic subject is not in a state of complete catatonia, then we must assume the existence of a mechanism of suppletion that has a compensatory function. This reasoning is fundamental to understanding cases of ordinary psychosis; the entire notion of stabilization in psychosis is, in a sense, premised on the kinds of catastrophic states evident in severe catatonia being *possible* in a psychotic structure, regardless of whether an individual ever experiences this kind of acute disturbance^28^. On the one hand, symptom severity is utilized in viewing catatonia as the most severe manifestation of schizophrenia; on the other, this “phenomenological” perspective is augmented by the claim that catatonia is characterized by the collapse of psychic reality due to the absence of a supplementary device. The absence of a supplementary device is catastrophic as the real remains unmediated by either the imaginary or the symbolic. The contrast between the severity of catatonic states and cases of ordinary psychosis highlights the variability of symptoms in psychotic structure: cases of ordinary psychosis demonstrate that sustaining psychic reality is possible in a psychotic structure even though the Name-of-the-Father is foreclosed. Miller's reference to symptom severity in catatonia constitutes a position from which the supplementary functions of the Name-of-the-Father may be developed. Thus, the second point concerning the neurosis/psychosis distinction follows on from this.

Miller (2006, 2009) is adamant that in order to understand ordinary psychosis, the functions of the Name-of-the-Father, as elaborated in neurosis, remain an important reference point for theorizing stabilization in psychosis. The CMB Name-of-the-Father aims to show how the psychotic subject has access to certain *functions* of the Name-of-the-Father even if this signifier is foreclosed. Throughout Lacan's teachings, the theory of the Name-of-the-Father became increasingly complex as more functions were attributed to it. Stevens (2007) description of the functions of the Name-of-the-Father in a neurotic structure provides a useful starting point for exploring this issue:

One needs “the Father who says no” as in “no smoking” i.e., the interdictor. This is the father who enforces a prohibition. This is the symbolic dimension of the father. However, if the father limits himself to this capacity… then it is primarily the imaginary aspect of the father that is present. The primacy of the imaginary register yields a specific aspect of enjoyment. It is jouissance of the body. One also needs “the Father who says yes.” This enabling dimension makes it possible for the subject to affirm his singularity and subsequently find his own way in the world. This is the father who makes it possible for the child to choose his own ideals. Finally, one also needs the inscription of a name, a unique name appropriate to the singularity of the subject. Thus, the Name-of-the-Father is also the transmission of the name. It is the symbolic inscription of the generations (p. 11).

These three functions of the Name-of-the-Father—castration, social identification, and naming—are associated with the reduction or tempering of jouissance. Thus, when working with the neurotic subject the Name-of-the-Father is deduced, in part, by observing its pacification effects; one clear indication of a neurotic structure is a limit to the “quantity” of jouissance (Miller, 2009). The neurotic subject's access to jouissance is limited by the cut of castration: in one sense, desire is a defense against jouissance. Lacan's statement that “castration means that jouissance has to be refused in order to be attained on the inverse scale of the Law of desire" (1966, p. 324) can be read in this light.

In psychosis, however, this limit to jouissance is not achieved via castration due the foreclosure of the Name-of-the-Father. Therefore, without these pacification effects of castration, jouissance may be invasive, delocalized and overwhelming. If “psychosis is essentially a disorder in the field of jouissance” ((Declerq, 2002), p. 102) then this occurs because there is no bar marking a limit to the subject's access to jouissance. Yet, despite the psychotic subject not having access to castration, pacification effects that would otherwise be linked to the Name-of-the-Father can be observed. The fact that pacifying effects *are evident* in cases of ordinary psychosis prompts the question of how stabilization occurs without the mechanism of castration. Consequently, mild forms of psychosis raises the question of how stabilization is possible. Miller's response is to ask: what functions of the Name-of-the-Father are evident in a supplementary device?

Miller's idea of the CMB Name-of-the-Father and its functions has continuity with Lacan's seminar on Joyce. The CMB Name-of-the-Father is an extension of (Lacan, 1975–1976) statement, made during the seminar on Joyce that *one can do without the Name-of-the-Father provided it is put to use*. On the one hand, the CMB Name-the-Father brings together the three distinct forms of suppletion in Lacan's theory of psychosis—imaginary identification, the delusion, and the sinthome; on the other, it is also emphasizes the importance of the *sinthome* in Lacan's later teachings. When surveying the literature on ordinary psychosis, it is clear that the emphasis on stabilization centers on imaginary identification and the sinthome. The vignette featuring Murielle leans toward the sinthome. Deffieux theorizes the case in terms of metonymy; that is, he traces how the emergence of a symptom was connected to the signifier “water” and the corporeal boundary delineated by the corset. According to Deffieux, symptoms can be tracked via how the signifier, *water*, is displaced into other signifiers that extract and localize body jouissance. The movement from *water cleansing rituals* through to the use of *hydrating cream* shows a mobility of jouissance and its gradual localization, which in turn, correlates with the disappearance of the invasive body pains. This process of displacement or metonymy appears quite different from Freud's description of “formations of the unconscious” in neurosis. That is, it is possible to assume that there is not repressed signifier underlying the movement of signifiers^29^. Moreover, the displacement does not occur in a delusional system. Deffieux's description highlights a decrease in painful body jouissance as a symptom coalesces around a series of signifiers linked to a bathing ritual that seems to reestablish corporeal unity. He also comments that her decision to join a group played an important *social identification* for her to create and maintain a group link. This last point is significant as the psychotic subject's difficulty creating and sustaining social links is a well-known feature of psychosis (i.e., anomie) and central to Lacan's claim that the psychotic subject is “outside” of discourse.

From my perspective, this case is useful in highlighting important aspects of ordinary psychosis. First, the case foregrounds a series of issues central to ordinary psychosis—triggering, mild psychosis, body disturbances and stabilization. As I have shown, while Verhaeghe's discussion of psychosis and actualpathology emphasizes similar themes, his reliance on the schizophrenia/paranoia dichotomy means that these issues are theorized primarily within the paranoia spectrum of psychosis. In contrast, as the vignette demonstrates the restitutive function of the sinthome in psychosis—without the delusional metaphor—then it requires a different response. The case is instructive in foregrounding core debates in the field of ordinary psychosis. A central problem, namely, how a sinthome can emerge to localize jouissance, remains to be sufficiently elaborated. Two aspects of the sinthome - the localisation of jouissance and social identification - are the focal point of investigation. In the case vignette, the emergence of a symptom is linked to Murielle's water cleansing “rituals” involving the body; this demonstrates that pacification effects associated with the Name-of-the-Father need to be theorized in conjunction with the signifier though in a manner that moves beyond the idea of the delusional metaphor. Moreover, the case also demonstrates how social identifications are central to understanding recovery in psychosis which supports the claim that “the sinthome is nothing other than the social bond for the subject” (Svolos, 2009, p. 3). Hence, Murielle creates a social link via participation with a writing group in the treatment facility. Deffieux indicates although the formation of a symptom correlated with the dissipation of painful hypochondriacal symptoms, it was the social identifications that facilitated a social link and it was this that lifted the negative symptoms. In summary, theorizing symptomatisation in psychosis beyond the scope of the delusional metaphor - which Deffieux does by focusing exclusively on metonymy - may shed light on cases where body phenomena and the signifier coalesce to create a stabilizing symptom in the subject's psychic economy.

## Conclusion

In contemporary Lacanian psychoanalysis, Verhaeghe's theory of psychosis and the Millerian field of ordinary psychosis provide distinct approaches to psychosis. Verhaeghe's theory, derived from Freud's idea of actual neurosis, psychoanalytic attachment theory and Lacan's notion of psychotic structure, engage a range of psychotic presentations in the schizophrenia/paranoia dichotomy. His theory of actualpathology in psychosis, which is characterized by the absence of positive/negative symptoms and the presence of subtle body disturbances is useful as it underscores the complex symptomatology of psychosis, an idea that is alarmingly absent in contemporary psychiatric discourse. Despite this utility, his theory of psychosis has several limitations. For example, distinguishing between the ideas of deviant mirroring styles and the mechanism of foreclosure in his description of drive disregulation remains difficult. In addition, Verhaeghe's discussion of compensatory mechanisms is too narrow as his treatment approach to psychosis recapitulates the schizophrenia/paranoia dichotomy. His approach to actualpathology in psychosis, where the analyst aims to modify body disturbances through the construction of symptoms and the emergence of secondary defenses, is essentially an attempt to transform schizophrenia into paranoia through the construction of a delusion. As such, there is insufficient attention given to the *variety* of compensatory mechanisms that may operate in psychosis. In contrast, the Millerian field of ordinary psychosis may provide a more useful approach to questions of stabilization in psychosis. It affirms the existence of a broad range of (often subtle) psychotic phenomena and supposes that an array of compensatory mechanisms can stabilize psychosis. By drawing extensively on Lacan's later teachings, theorists in the field of ordinary psychosis have begun to reformulate Freud's thesis of loss and restitution in psychosis beyond the construction of a delusion by supposing that a symptom with a stabilizing function may emerge in a variety of forms. While this focus on stabilization and compensatory mechanisms in psychotic structure is under-theorized, it does provide a useful theory for reviving the idea of mild psychosis. In summary, the field of ordinary psychosis, in broadening the category of psychosis and through placing greater emphasis on the idea of untriggered psychosis and the sinthome, has greater utility than Verhaeghe's theory of psychosis, which remains anchored in the narrower schizophrenia/paranoia dichotomy.

### Conflict of interest statement

The author declares that the research was conducted in the absence of any commercial or financial relationships that could be construed as a potential conflict of interest.

## Footnotes

^1^I use the term *onset* when referring to the notion of the *first psychotic episode*; in contrast, I use the phrase *triggering events* when referring to the emergence of psychotic episodes occurring after the original *onset*.

^2^A third clinical structure, *perversion*, is also utilized in Lacanian theory (Dor, 1997) but will not be discussed here due to its marginal status in the clinical field (Fink, 1997) and ongoing doubt over its nosological status (Miller, 2009).

^3^Verhaeghe's claim that ordinary psychosis will lead to confusion between psychoanalysts and other mental health professionals is misguided. As Miller (2009) himself has stated, using the term ordinary psychosis is not an excuse to ignore the modern psychiatric clinic, rather, the term invites clinicians to reengage with the knowledge obtained by the “masters” of classical psychiatry such as Kraepelin, Bleuler, de Clérambault and others, in the attempt to mitigate the reductive idea of psychosis explicated in contemporary psychiatry.

^4^Lacan states:
I remind you that at the end of the observation on the Schreber case, which is his major text concerning the psychoses, Freud traces out a watershed, as it were, between paranoia on the one hand and on the other everything he would like, he says, to be called paraphrenia, which exactly covers the field of the schizophrenias. This is a necessary reference point for the intelligibility of everything we shall subsequently have to say—for Freud the field of the psychoses divides in two (Lacan, 1993, p. 4).

^5^However, Lacan's comment on the importance of imaginary identification as a mechanism of stabilization encountered in untriggered schizophrenia, derived in part from his commentary on Deutsch (1942), was also introduced at this time and remains a significant component in the treatment of psychosis.

^6^Vanheule's (2011) The subject of psychosis: a Lacanian perspective for a useful discussion of the delusional metaphor and the paternal metaphor.

^7^The reader should be informed that Verhaeghe's actualpathology/psychopathology is not restricted to psychosis and is superimposed on to Lacan's three clinical structures: neurosis, psychosis, and perversion.

^8^Verhaeghe (2004) uses the term “the actualpathological position of the subject” when referring to the clinical phenomena outlined by Freud's idea of the actual neuroses; moreover, like Freud, his approach to actualpathological states moves across the diagnostic spectrum: neurosis, psychosis, and perversion. Although my discussion focuses on actualpathological states in psychosis, the mechanism underlying actualpathology—the failure of the Other to adequately mirror, and hence, modulate the subject's endogenous drive tension—is applicable to neurosis, psychosis, and perversion.

^9^The DSM-IV-TR's (2000) Generalized anxiety disorder has its nosological antecedents in Freud's theory of the actual neuroses (Tyrer and Baldwin, 2006).

^10^Freud developed the theory of actual neurosis to address instances where unconscious processes did not function in the emergence of clinical phenomena. In the Introductory lectures to psychoanalysis he states:
But the symptoms of the “actual” neuroses—intracranial pressure, sensations of pain, state of irritation in an organ, weakening or inhibition of a function—have no “sense,” no psychical meaning. They are not only manifested predominately in the body… but they are also themselves entirely somatic processes, in the generating of which all the complicated mental mechanisms we have come to know are absent (Freud, 1916–1917, p. 387). From one point of view, Freud maintains that somatic phenomena are devoid of psychological meaning, as the mechanisms of displacement and condensation do not operate in these phenomena. For example, if a headache were a neurotic conversion symptom then the analysand's associations, memories and unconscious fantasies would eventually illuminate the meaning of the symptom.

^11^Freud's theory of the actual neuroses has been particularly influential in the area of “psychosomatic” theories of illness; see McDougall and Coen (2000), Aisemberg and Aisenstein (2004); Taylor (2003); Verhaeghe (2004), and Verhaeghe et al. (2007) for a discussion of these issues.

^12^See Vanheule (2011) for a good discussion of Lacan's idea of the object *a* in psychosis.

^13^Verhaeghe explicitly draws on the idea of infantile helplessness and Freud's classic description of drives in the Three essays. Here Freud states:
The concept of instinct is thus one of those lying on the frontier between the mental and the physical. The simplest and likeliest assumption as to the nature of instincts would seem to be that in itself an instinct is without quality, and, so far as mental life is concerned, is only to be regarded as a measure of the demand made upon the mind for work. What distinguishes the instincts from one another and endows them with specific qualities is their relation to their somatic sources and to their aims (1905, p. 67).

^14^Verhaeghe (2004) uses the term “the psychopathological position of the subject” when referring to what Freud (1894) called the “neuro-psychoses of defense”; this includes hysterical neurosis, obsessional neurosis, anxiety hysteria and paranoia. What these entities have in common is that primary process mechanisms are active determinants in symptom formation.

^15^Verhaeghe appears to uses the term the Other to connote both the small other (i.e., the individuals ego to ego relation with others as such) and the Other as language and social order.

^16^Fonagy et al. claim that this will likely create a distorted perception of the self-state, a distortion that undermines self-development similar to Winnicott's notion of the “false self” (2002).

^17^See Lacan's The instance of the letter in the unconscious or reason since Freud for a discussion of the metaphorical structure of the symptom.

^18^The idea here is that that articulation of signifiers will have a pacifying effect on jouissance.

^19^The series of conferences are published as Conversation d'Arcachon, Convention d'antibes and Conciliabule d'Anger.

^20^See Vanheule (2011) for a slightly different reading on the status of the neurosis/distinction in Lacan's later teachings.

^21^I use the phrase “triggering events” when referring to post-onset psychotic episodes. In contrast, I use the term “onset” when referring to untriggered psychosis and the first psychotic episode.

^22^Bleuler's idea of latent schizophrenia is coextensive with the DSM personality disorder “schizotypal disorder” (Kety, 1985).

^23^Cenesthesias were described well-before Huber (i.e., De Clérambault) but his work has been central in attempts to create a diagnostic category for this form of schizophrenia.

^24^Lacan's theory of imaginary identification in cases of untriggered psychosis was derived, in part, from Deutsch's (1942) theory of the “as if” phenomena observed in schizophrenia.

^25^Although this is not Stevens' case, he provides a detailed description of the patient's history and symptomatology centered on the fibromyalgia.

^26^As Gault states,
the subjects' efforts to defend themselves against the real had brought them to elect a part of their body as painful and to attempt to raise this pain to the status of a symptom addressed to an analyst. Although these were phenomena related purely to the signifier's effect on the body as a living organism, these “neo-conversions” do not, in our opinion, belong to the register of the hysterical structure (2008, p. 2).

^27^Lacan's classical theory of psychosis refers to the essays and seminars in the 1950' s where the foreclosure of the Name-of-the-Father was first introduced (1958, 1993).

^28^Again, the importance of Lacan's unitary theory of psychosis cannot be underestimated because different variants of psychosis are assumed to share the same fundamental mechanism, and continuity between these different forms will be evident in many cases. However, determining the different mechanisms operating in diverging forms of psychosis such as schizophrenia, paranoia and melancholy, remains an important theoretical task.

^29^For example, in Studies on hysteria (1893–1895) the case of Fräulein Elizabeth von R. shows how a conflict concerning the emergence of erotic ideas was pivotal in the development of conversion symptoms. In this particular case, Freud states that Elizabeth's conversion symptom—a localized pain to her right upper thigh—first developed when
the circle of ideas embracing her duties to her sick father came into conflict with the content of the erotic desire she was feeling at the time. Under pressure of lively self-reproaches she decided in favor of the former, and in doing so brought about her hysterical pain (1893–1895, p. 164). Localization of the hysterical conversion symptom to her right thigh correlates with the place her father would rest his foot when Elizabeth was bandaging his ankle during his convalescence; these memories provided the “content” for the dissimulation of erotic wishes via the construction of a symptom (Freud and Breuer, 1893–1895). Although this is only a small segment of this case, the point is that the hysterical conversion symptom emerged due to repression; in this case, repression functioned as a defense against acknowledging ideas that created psychical conflict.
